# Multiple intracranial enlarging dissecting aneurysms: a case report

**DOI:** 10.1186/s12883-023-03303-6

**Published:** 2023-07-12

**Authors:** Yingbin Li, Xiaoxin Bai, Huai Tu, Zhimin Zou, Yan Huang, Jun Cai

**Affiliations:** 1 Diagnosis and Treatment Center of Encephalopathy, The Second Affiliated Hospital of Guangzhou University of Chinese Medicine, Guangzhou, 510120 China; 2 Department of Cerebrovascular Surgery, Guangdong Provincial Hospital of Chinese Medicine, Hospital of Guangzhou University Mega Center, Guangzhou, 510006 China; 3 Department of Neurosurgery, Hospital of Guangzhou Higher Education Mega Center, Guangdong Provincial Hospital of Chinese Medicine, 55 Neihuan Xi Road, Guangzhou, 510006 Guangdong China

**Keywords:** Cerebral aneurysm, Cerebral ischemia, Endovascular treatment, Dissecting aneurysm, Subarachnoid hemorrhage

## Abstract

**Background:**

Cases of multiple cerebral aneurysms are rare. In this case report, we describe a male patient with multiple, enlarging, and ruptured aneurysms. The two aneurysms were believed to be dissecting aneurysms.

**Case description:**

A 47-year-old man presented with left limb paralysis. Magnetic resonance imaging revealed a cerebral infarction. Digital subtraction angiography (DSA) identified an aneurysm and occlusion in the right middle cerebral artery (MCA). The MCA aneurysm was remarkably enlarged on the eighth day after cerebral ischemia and was treated using endovascular techniques. Two weeks after the endovascular treatment, the patient experienced a severe headache and became comatose, and a subarachnoid re-hemorrhage was confirmed. The fourth DSA revealed an enlarging dissecting aneurysm in the posterior cerebral artery. The patient died without further treatment.

**Conclusion:**

Some dissecting aneurysms rapidly enlarge and rupture.

**Supplementary Information:**

The online version contains supplementary material available at 10.1186/s12883-023-03303-6.

## Background

Cerebral aneurysm formation is postulated to occur via the following mechanisms: (1) initial apoptosis of vascular smooth muscle cells within the vessel wall and disruption of the internal elastic lamina. (2) Collagen fiber reconstitution due to the resulting shift in tensile forces, leading to subsequent collagen and/or elastin degradation and breakdown, and therefore vessel wall remodeling [1, 2]. In a meta-analysis including data from nearly 5000 unruptured aneurysms, 9% enlarged over a mean duration of 2.8 years [3]. In the present study, we report the case of a male patient with a middle cerebral artery (MCA) aneurysm who developed an enlarging dissecting aneurysm located in the posterior cerebral artery (PCA) in an extraordinarily short time period. De novo aneurysms account for up to 1.8% of aneurysmal subarachnoid hemorrhage (SAH) cases annually [4]. Owing to their rare incidence and lack of case reports, their formation, progression, and development remain unclear [5, 6]. Most newly founded aneurysms progress gradually and remain stable for a certain period [7]. Here, we present a case of cerebral artery occlusion and a dissecting aneurysm in the M1 section of the right MCA. The aneurysm progressed rapidly (within 7 days) prior to endovascular treatment. Two weeks after left MCA aneurysm coiling, the patient developed SAH again. Digital subtraction angiography (DSA) imaging confirmed the presence of an enlarging dissecting aneurysm in the P1 section of the left PCA. The patient died of recurrent SAH.

## Case description

**Fig. 1:**
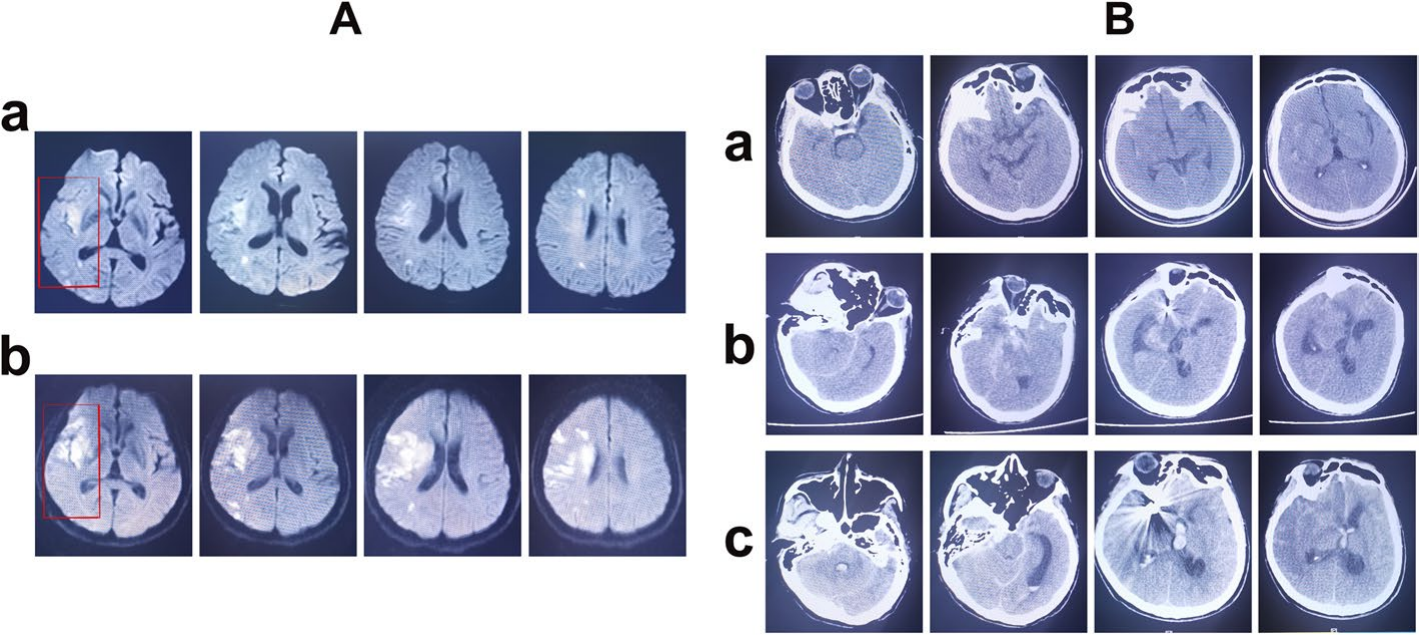
Magnetic resonance and computed tomography images. **A** Panel a: Diffuse-weighted magnetic resonance imaging sequence acquired on the day of admission. Panel b: Diffuse-weighted magnetic resonance imaging sequence acquired on the day after admission. The rectangular area displays a cerebral infarction lesion located in the left watershed region. **B** Panel a: Computed tomography image taken on the day of admission. Panel b: Computed tomography image taken on the day the patient suffered a new acute subarachnoid hemorrhage. Panel c: Computed tomography image taken during the final digital subtraction angiography

A 47-year-old man with a history of hypertension and diabetes mellitus presented to the emergency room with sudden-onset hemiplegic paralysis on the left side, which had persisted for 3 h. Acute cerebral ischemia was confirmed by magnetic resonance imaging (MRI; Fig. 1A). The patient suffered from hemiparesis, with a score of 2 points according to the National Institutes of Health Stroke Scale (NIHSS), as evaluated by two neurologists.

**Fig. 2:**
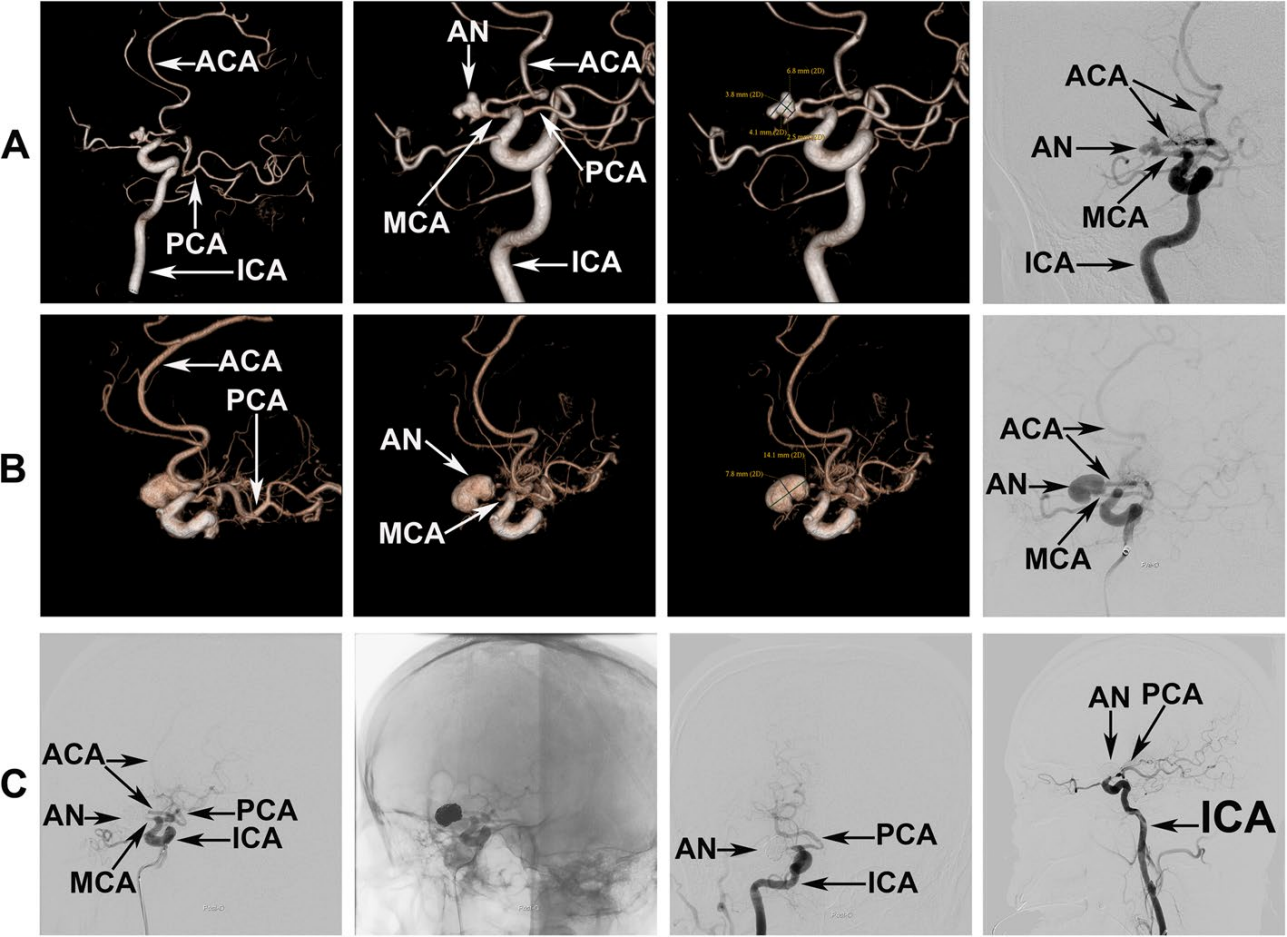
Images of the first two digital subtraction angiography procedures. **A** Images of the first digital subtraction angiography on the day of admission, revealing a dissecting aneurysm in the occluded right middle cerebral artery. **B** Images of the second digital subtraction angiography on the day the trapping of the dissecting aneurysm in the right middle cerebral artery was scheduled, indicating that the dissecting aneurysm had progressed remarkably. **C** Images of the second digital subtraction angiography, revealing that the dissecting aneurysm located in the right middle cerebral artery was completely trapped. ACA, anterior cerebral artery; AN, aneurysm; ICA, internal cerebral artery; MCA, middle cerebral artery; PCA, posterior cerebral artery

However, computed tomography (CT) performed before the MRI revealed a subacute SAH (Fig. 1B); thus, intravenous thrombolysis with a recombinant tissue plasminogen activator could not be performed. DSA performed after CT and MRI revealed a dissecting aneurysm in the M1 section of the MCA and distal occlusion further along the aneurysm (Fig. 2A). Based on the DSA images, we postulated that recanalization of the occluded MCA was too high risk. Furthermore, we considered extracranial-intracranial bypass; however, this was declined by the patient and his family. Thus, conservative treatment was administered with dual antiplatelets (100 mg aspirin and 75 mg clopidogrel daily) and lipid-lowering therapy (40 mg atorvastatin calcium tablet daily), without endovascular treatment. We planned to embolize the dissecting aneurysm in the right MCA one week later.

Unfortunately, the patient experienced more severe neurological deficits within 24 h, resulting in a new NIHSS score of 12 points. Repeated CT scans excluded intracranial hemorrhage or hemorrhagic transformations. Additional cerebral ischemia was detected using diffusion-weighted imaging (DWI) (Fig. 1A-b). We continued the conservative therapeutic strategy. Although hypertension and diabetes mellitus were confirmed by biomedical tests, arterial pressure and blood glucose levels were normal. The neurological function of the patient remained stable. All Glasgow Coma Scale and NIHSS scores are presented in Supplementary Data 1A, and arterial pressure monitoring data are presented in Supplementary Data 1B.

**Fig. 3:**
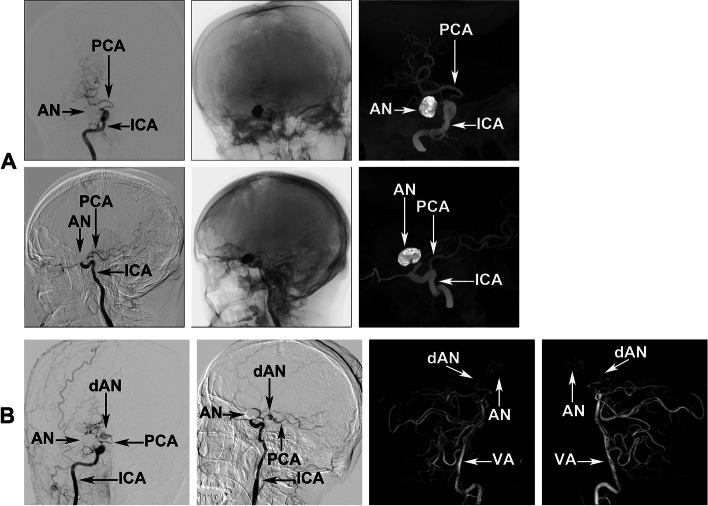
DSA images following right MCA aneurysm trapping. **A** Images of the third DSA procedure, performed 9 days after the right MCA aneurysm had been coiled, showing that the coiled aneurysm was completely sealed off. Three-dimensional ICA angiography and left vertebral artery angiography images confirmed the P1 section of the right PCA as normal at that time. **B** Images of the fourth DSA procedure, which was performed after a repeat subarachnoid hemorrhage (15 days after coiling of the right MCA aneurysm). The two- and three-dimensional DSA images reveal an enlarging dissecting aneurysm located in the P1 section of the right PCA. ACA, anterior cerebral artery; AN, aneurysm; dAN, de novo aneurysm; DSA, digital subtraction angiography; ICA, internal cerebral artery; MCA, middle cerebral artery; PCA, posterior cerebral artery

One week later, endovascular treatment was administered to the M1 aneurysm according to the therapeutic procedure. During this procedure, we observed that the dissecting aneurysm had enlarged significantly, with the volume having more than tripled in one week (Fig. 2B). The endovascular procedure was completed satisfactorily (Fig. 2C) and the patient underwent neurological rehabilitation. We performed a second DSA nine days after the endovascular procedure, which confirmed successful treatment of the MCA aneurysm (Fig. 3A and Supplementary Data 2). A CT scan performed before DSA revealed that the SAH was barely detectable (Supplementary Data 3).

**Fig. 4:**
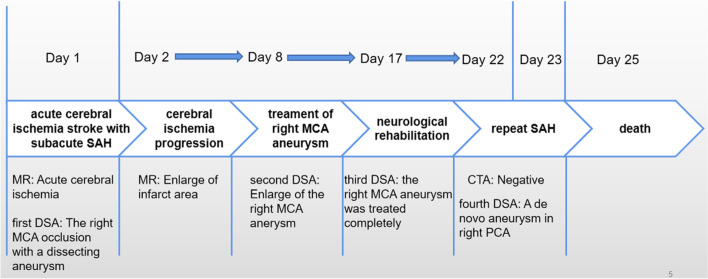
A schematic diagram detailing the course of the multiple aneurysms and the treatment modality employed

However, the patient experienced a severe headache 5 days after the repeat DSA. CT confirmed a new acute SAH (Fig. 1B-b). CT angiography was performed immediately, which revealed no evidence of re-rupture of the treated aneurysm or other new aneurysms (Supplementary Data 4). To exclude other hemorrhagic cerebral vascular diseases, such as arterial venous malformation and carotid-cavernous sinus fistula, we performed a fourth DSA on the day after the hemorrhage recurrence. The DSA revealed that an enlarging dissecting aneurysm had been detected in the right PCA (Fig. 3B), which had not been seen previously. In addition, a new SAH was confirmed on CT during DSA (Fig. 1B-c). We intended to perform endovascular treatment by trapping the enlarging dissecting aneurysm and parent artery; however, the patient’s family declined further invasive treatment. Thus, we administered conservative treatment to the patient who died two days later. A schematic diagram (Fig. 4) details the course of the multiple aneurysms and the treatment modality employed in the present case.

## Discussion and conclusions

Dissecting intracranial aneurysms are always located in the anterior circulation, mostly in the internal carotid artery. Young patients (those aged 60 years and under) with dissecting aneurysms experience SAH or cerebral ischemia [8]. However, in this case, the same patient suffered from both SAH and cerebral ischemia, which, to the best of our knowledge, has not been previously reported. Anticoagulant or antiplatelet therapies prevent cerebral ischemic stroke in patients with dissecting aneurysms. Moreover, there is no difference in ischemic or hemorrhagic events between patients treated with anticoagulants and those treated with aspirin [9]. In the present case, we treated the patient with dual antiplatelets; however, the patient experienced progressive ischemic cerebral apoplexy. The endovascular treatment strategy for a dissecting cerebral aneurysm involves reconstruction of the normal vascular endothelial structure with stents, including flow diverters [10]. However, this treatment strategy was not suitable in the present case because of the MCA occlusion. In addition, the use of a flow diverter was not indicated for the dissecting aneurysm located in the right PCA because the dissecting aneurysm ruptured within a short period.

The incidence of de novo aneurysms has been reported to range from 0.1% to 2% per year [11–14], and most newly founded aneurysms appear in the anterior circulation [15]. According to a retrospective study in Finland that included 56 newly founded aneurysms in 42 patients from a total population of 1419 patients with intracranial aneurysms, the proportion of de novo aneurysms in the posterior cerebral circulation, including the basilar and vertebral arteries, was 19.4% (11/56) [15]. The most commonly reported de novo aneurysms in the posterior or anterior cerebral circulation were saccular. However, newly dissecting aneurysms are rare; a ruptured dissecting aneurysm was analyzed in 2002, and a fusiform aneurysm was reported in 1991 [16, 17]. In the present case, the aneurysm was located in the M1 section of the right MCA, and the distal section following the aneurysm was occluded. Therefore, the aneurysm was considered to be dissecting. From the first three DSA procedures, we could clearly exclude any aneurysm located in the P1 section of the right PCA, which was detected in the fourth and final DSA procedure. Thus, the aneurysm located in P1 of the right PCA was a newly founded aneurysm. Based on morphology, the newly founded aneurysm was suspected to be a dissecting aneurysm. Moreover, the newly founded dissecting aneurysm developed and ruptured within 1 week. To the best of our knowledge, this is the first report of a newly founded enlarging dissecting aneurysm that ruptured so soon after its development.

The etiology of these newly founded aneurysms remains unclear. Internal elastic lamina defects are an important etiological feature of aneurysm development [17]. Hemodynamic forces are another critical factor in inducing de novo aneurysms [18]. In the present case, P1 morphology was detected in the first DSA procedure; however, the physiological appearance was normal. We postulate that the hemodynamic forces at this location may have increased substantially following endovascular treatment. Thus, the two aforementioned etiological factors may have induced the development of the enlarging dissecting aneurysm located at P1 of the right PCA. Other possible contributing factors, such as hypertension, atherosclerosis, and smoking [11, 17, 19], were also present in this case. A systematic review and meta-analysis that included 14,968 patients with aneurysms from 35 studies reported that the rupture timing in most de novo aneurysm cases was more than 10 years [11]. Another retrospective clinical study that analyzed 56 de novo aneurysms in 1419 patients, reported a rupture rate of approximately 16% (9/56) in a median of 11.2 years [15]. In contrast, in the present case, the enlarging dissecting aneurysm ruptured at least twice within 72 h.

The pathological and radiological features of dissecting cerebral aneurysms were described by Yonas et al. in 1977 [20]. Dissecting cerebral aneurysms are widely considered an important cause of cerebral ischemic stroke in young adults, although this is a relatively rare occurrence [21, 22]. Dissecting cerebral aneurysms are responsible for 1–2% of cerebral ischemic strokes, although this rises to 10–25% in younger patients [23].

In addition, the clinical features of dissecting cerebral aneurysms are heterogeneous [22]. In the present case, the patient presented with cerebral ischemia in the right hemisphere following an SAH. Therefore, the therapeutic strategy for this rare and contradictory case was complex. A single therapeutic strategy would not be able to treat both the dissecting aneurysm and the occluded artery. In this case, the optimal strategy was acute extracranial-intracranial bypass and trapping of the dissecting aneurysm in one stage. However, this approach was declined by both the patient and his family members. Therefore, the patient received drug therapy and intensive care. However, the neurological deficits of the patient became more severe the following day. The relatives of the patient requested more aggressive treatment; however, extracranial-intracranial bypass was no longer an option owing to the massive infarct area revealed by DWI-MRI. Confluent infarct volume in the subcortex is reportedly associated with hemorrhage transformation following cerebral ischemia [24]. Therefore, different strategies were selected. First, we planned to occlude the MCA dissecting aneurysm and parent artery endovascularly one week after the ischemic stroke. Second, we planned to perform direct and indirect extracranial-intracranial bypass one month after the ischemic stroke. The patient and his family agreed to this therapeutic strategy. According to previous studies, quantitative evaluation of cerebral blood flow should be used to monitor the size of developing infarcts [25], and CT perfusion (CTP) can be used to predict the early malignant course of progressive cerebral ischemia [26]. In the present case, we used transcranial Doppler instead of CTP to detect regional cerebral blood flow. If quantitative monitoring of regional blood flow had been performed to monitor regional cerebral blood flow changes, it might have indicated the occurrence of progressive cerebral ischemia. We aimed to improve cerebral perfusion using conservative treatment. Most importantly, if we had used CTP to monitor regional cerebral blood flow in the right MCA, the changes in the enlarging dissecting aneurysm could have been detected through changes in regional blood flow. The changes in regional cerebral blood flow resulted in progressive cerebral ischemia, which disabled the patient. Using DSA, we confirmed the rapid progression of the first dissecting aneurysm located in the right MCA and verified the transitory formation of the second dissecting aneurysm located in the right PCA. However, the optimal timing for repeated DSA is difficult to determine. In the first three DSA procedures, the P1 section of the right PCA was carefully inspected using internal carotid angiography. Due to the existence of the right posterior communicating artery in the patient, we could perform angiography of the right PCA through the right internal artery. Some studies have indicated that cerebral vasospasm resulting from SAH may hinder the diagnosis of a cerebral aneurysm [27, 28]. However, in the present case, transcranial Doppler sonography was performed twice to monitor blood velocity in the infarct area. No evidence of cerebral vasospasm was observed in the adjacent area, including the right PCA-supplying area.

## Supplementary Information

**Additional file 1: Supplementary Data 1.** Neurological function and arterial pressure monitoring during hospitalization. A Line graphs displaying NIHSS and GCS scores of the patient at all stages of hospitalization. B Line graphs showing systolic and diastolic blood pressures of the patient during hospitalization. GCS, Glasgow Coma Scale; NIHSS, National Institutes of Health Stroke Scale.

**Additional file 2: Supplementary Data 2.** DSA images displaying morphological changes in the dissecting aneurysm located in the right MCA before and after the endovascular procedure. A DSA images showing the aneurysm morphology before and after endovascular treatment in the second DSA procedure. B DSA images showing aneurysm morphology after the endovascular procedure in the third DSA procedure. Posterior cerebral circulation angiography was performed through the left vertebral artery. No signs of an aneurysm were detected in the right PCA during the fourth DSA procedure. AN, aneurysm; dAN, de novo aneurysm; DSA, digital subtraction angiography; ICA, internal cerebral artery; MCA, middle cerebral artery; PCA, posterior cerebral artery.

**Additional file 3: Supplementary Data 3.** Computed tomography performed after coiling of the middle cerebral artery aneurysm revealing that the subarachnoid hemorrhage was barely distinguishable.

**Additional file 4: Supplementary Data 4.** Computed tomography angiography images immediately after re-hemorrhage in the subarachnoid cavum. No signs of aneurysm recurrence or de novo aneurysms were observed in these images.

## Data Availability

The datasets used and/or analyzed during the current study are available from the corresponding author on reasonable request.

## Abbreviations

CT: Computed tomography
CTP: Computed tomography perfusion
DSA: Digital subtraction angiography
DWI: Diffusion-weighted imaging
MCA: Middle cerebral artery
MRI: Magnetic resonance imaging
NIHSS: National Institutes of Health Stroke Scale
PCA: Posterior cerebral artery
SAH: Subarachnoid hemorrhage
