# Supplementary figures and images for: Multiple intracranial enlarging dissecting aneurysms: a case report
Source: BMC Neurol. 2023 Jul 12;23:265. doi: 10.1186/s12883-023-03303-6 (PMC10337085; doi:10.1186/s12883-023-03303-6)

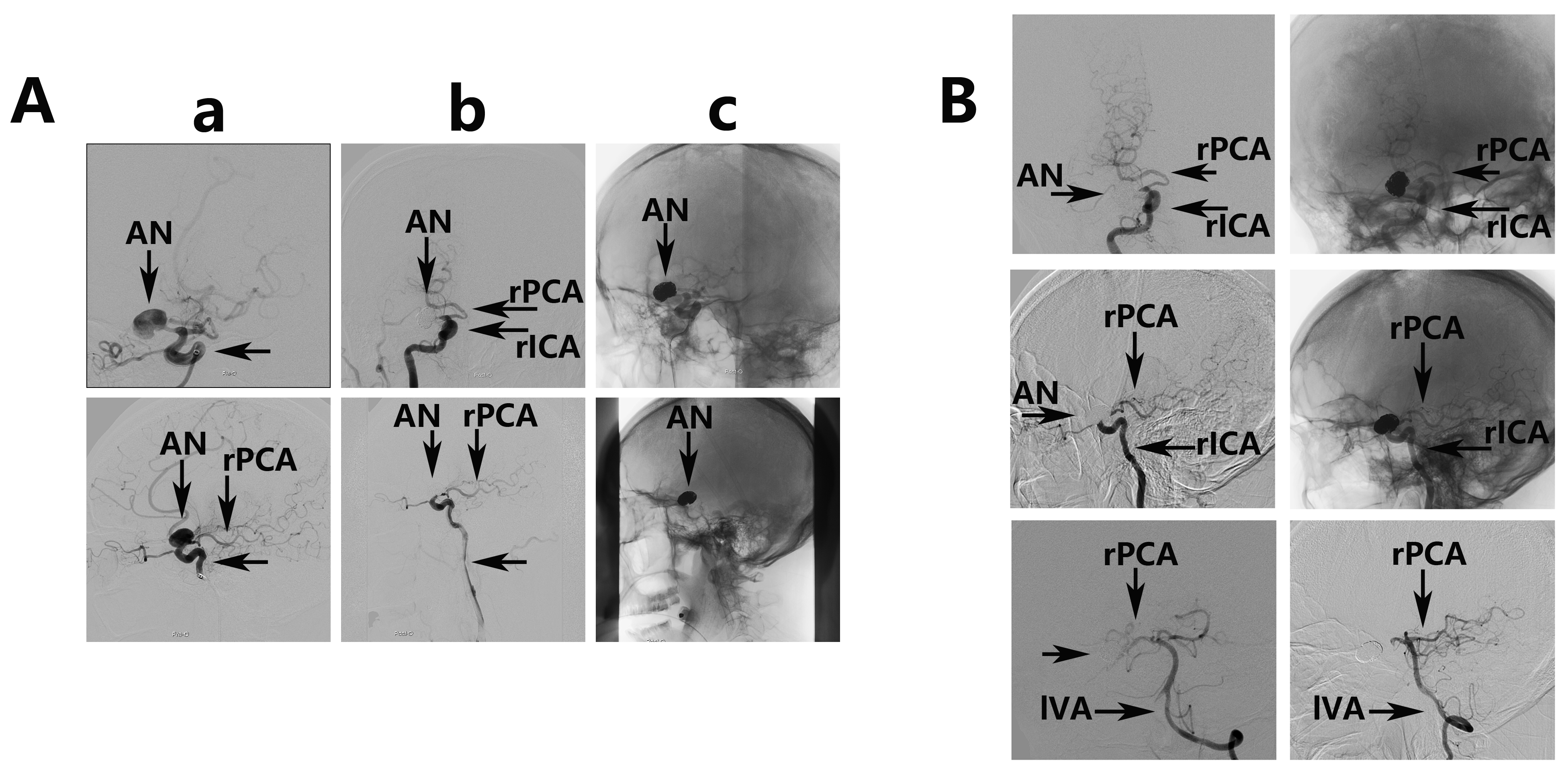

Supplement: Supplementary file 1 — Additional file 1: Supplementary Data 1. Neurological function and arterial pressure monitoring during hospitalization. A Line graphs displaying NIHSS and GCS scores of the patient at all stages of hospitalization. B Line graphs showing systolic and diastolic blood pressures of the patient during hospitalization. GCS, Glasgow Coma Scale; NIHSS, National Institutes of Health Stroke Scale. [file 12883_2023_3303_MOESM1_ESM.tif]

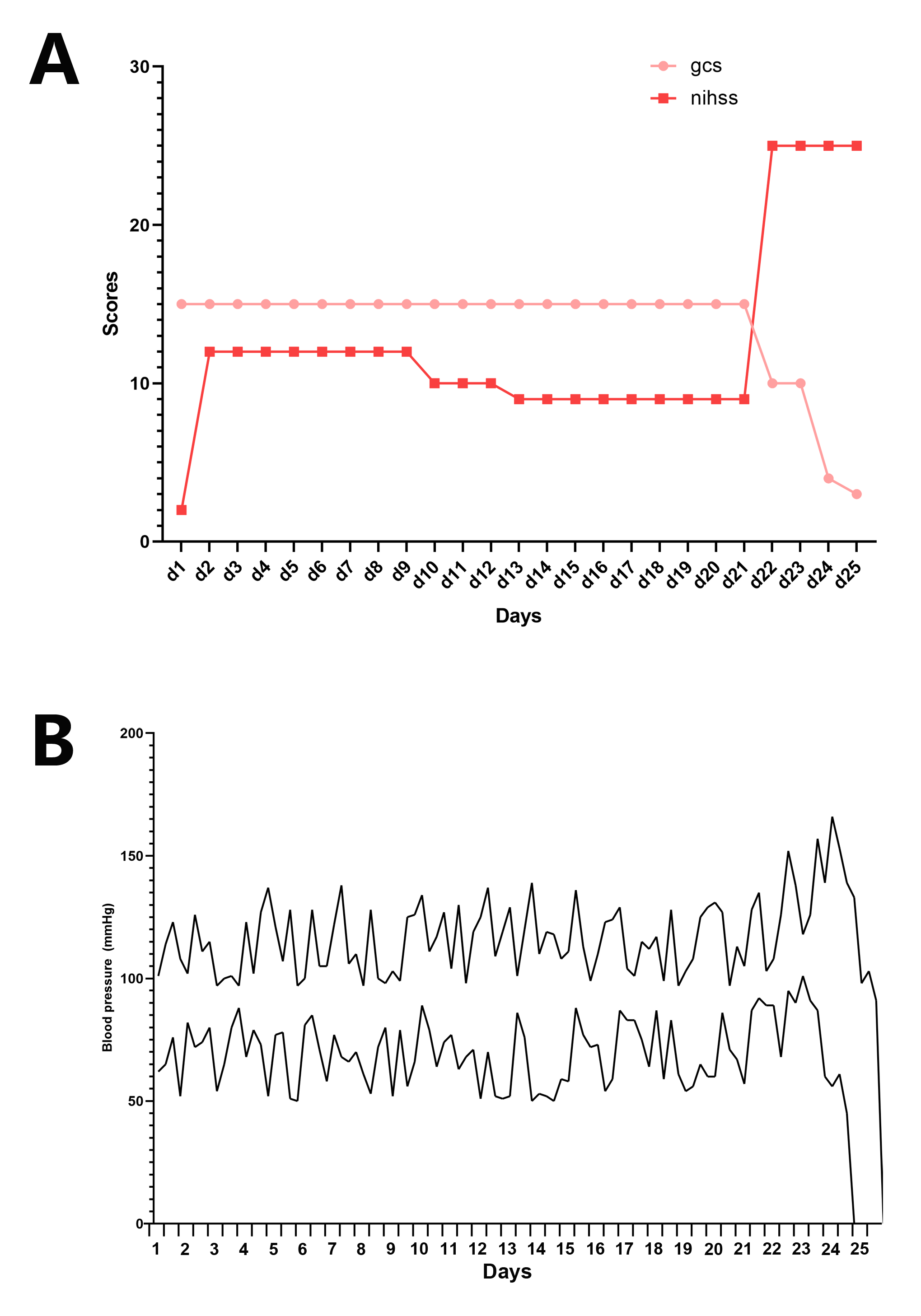

Supplement: Supplementary file 2 — Additional file 2: Supplementary Data 2. DSA images displaying morphological changes in the dissecting aneurysm located in the right MCA before and after the endovascular procedure. A DSA images showing the aneurysm morphology before and after endovascular treatment in the second DSA procedure. B DSA images showing aneurysm morphology after the endovascular procedure in the third DSA procedure. Posterior cerebral circulation angiography was performed through the left vertebral artery. No signs of an aneurysm were detected in the right PCA during the fourth DSA procedure. AN, aneurysm; dAN, de novo aneurysm; DSA, digital subtraction angiography; ICA, internal cerebral artery; MCA, middle cerebral artery; PCA, posterior cerebral artery. [file 12883_2023_3303_MOESM2_ESM.tif]

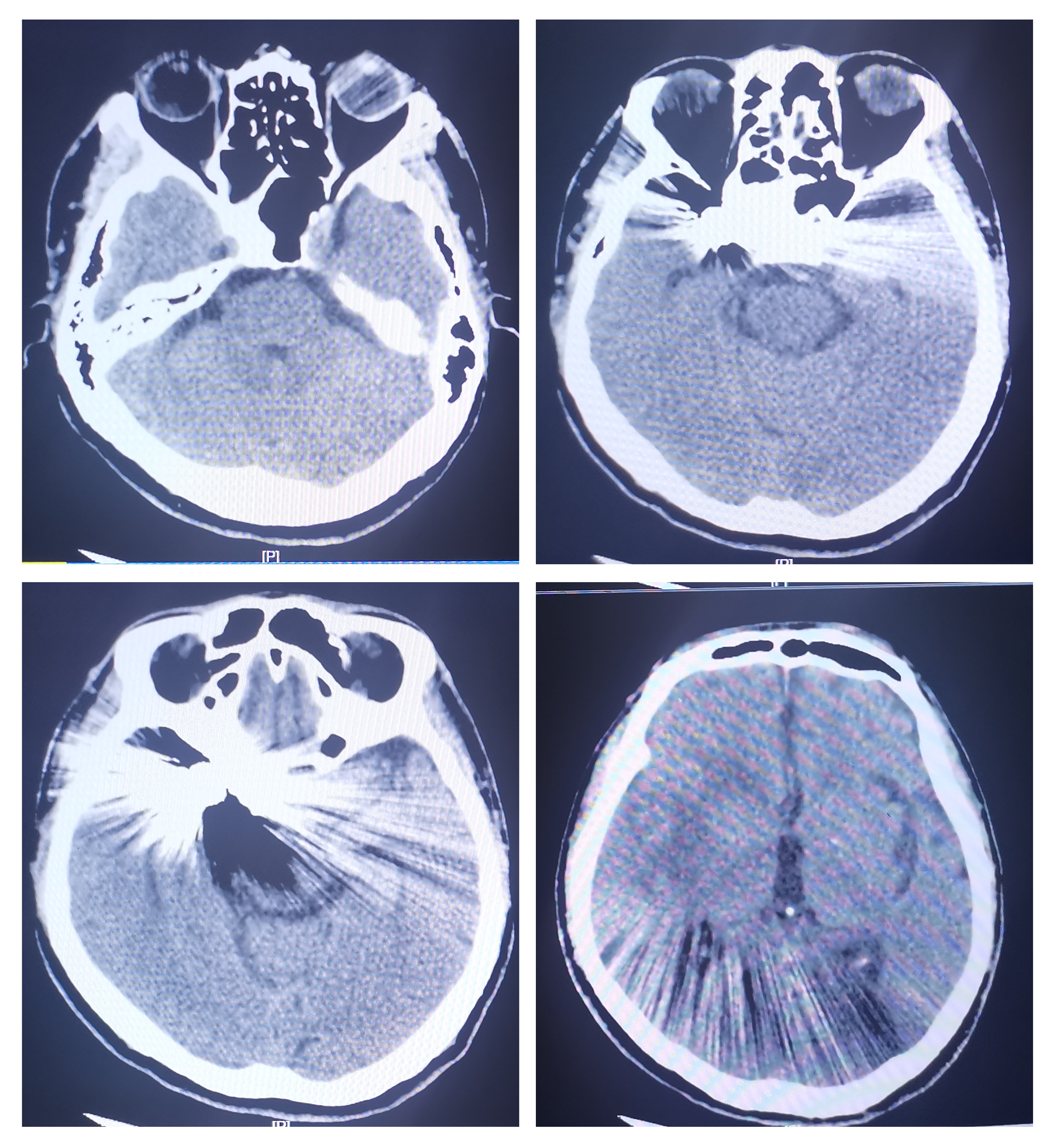

Supplement: Supplementary file 3 — Additional file 3: Supplementary Data 3. Computed tomography performed after coiling of the middle cerebral artery aneurysm revealing that the subarachnoid hemorrhage was barely distinguishable. [file 12883_2023_3303_MOESM3_ESM.tif]

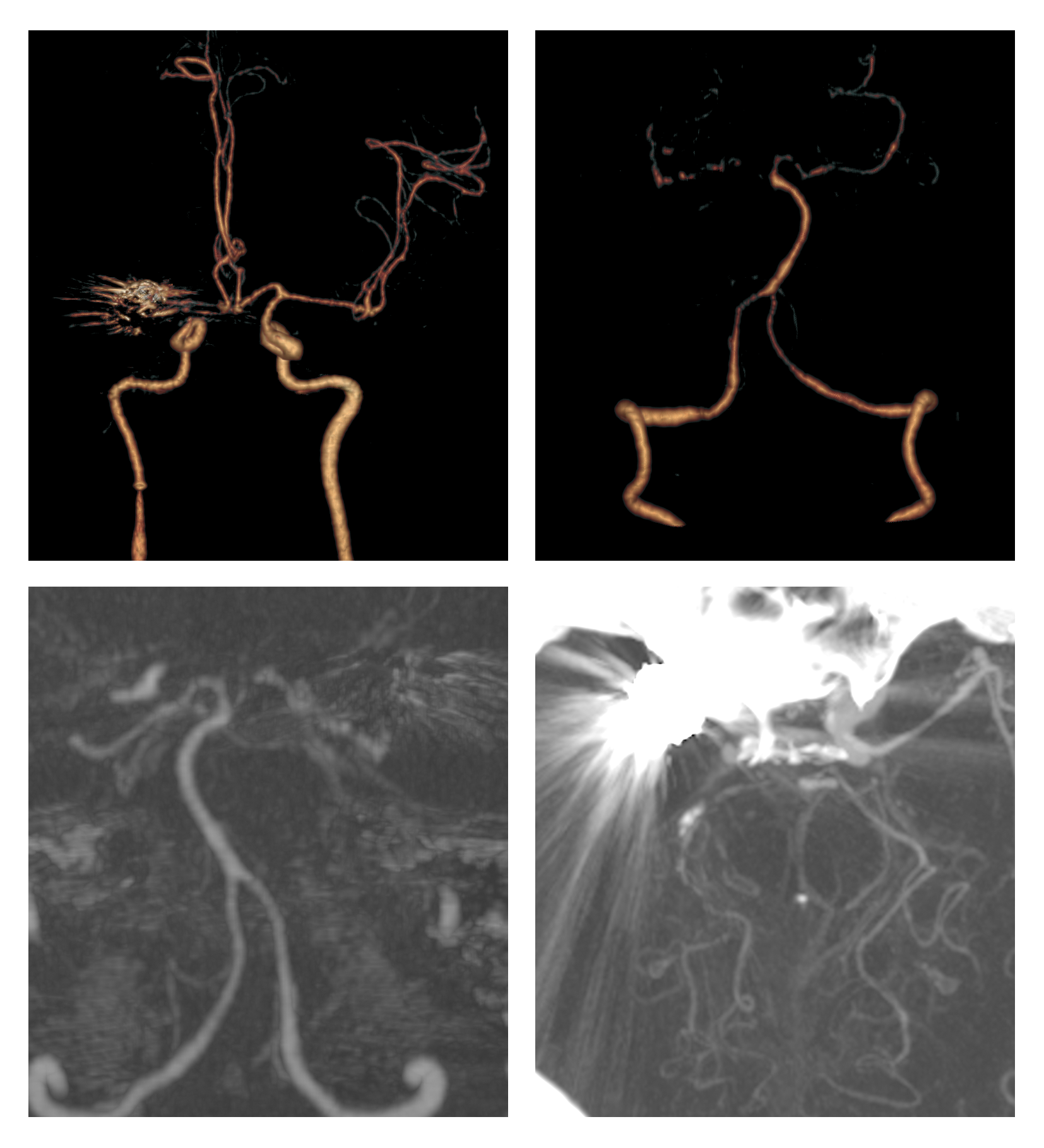

Supplement: Supplementary file 4 — Additional file 4: Supplementary Data 4. Computed tomography angiography images immediately after re-hemorrhage in the subarachnoid cavum. No signs of aneurysm recurrence or de novo aneurysms were observed in these images. [file 12883_2023_3303_MOESM4_ESM.tif]
